# Clinical presentation of abdominal tuberculosis in HIV seronegative adults

**DOI:** 10.1186/1471-230X-5-21

**Published:** 2005-06-21

**Authors:** Cengiz Bolukbas, Fusun F Bolukbas, Tulin Kendir, Remzi A Dalay, Nihat Akbayir, Mehmet H Sokmen, Ali T Ince, Mithat Guran, Erkan Ceylan, Guray Kilic, Oya Ovunc

**Affiliations:** 1 Department of Internal Medicine, Gastroenterology Division, Faculty of Medicine, Harran University, Sanliurfa, Turkey; 2 Gastroenterology Clinic, Haydarpasa Numune Training and Research Hospital, Istanbul, Turkey; 3 Gastroenterology Clinic, Sisli Etfal Training and Research Hospital, Istanbul, Turkey; 4 General Surgery Clinic, Haydarpasa Numune Training and Research Hospital, Istanbul, Turkey; 5 Department of Chest Diseases, Faculty of Medicine, Harran University, Sanliurfa, Turkey; 6 Pathology Unit, Haydarpasa Numune Training and Research Hospital, Istanbul, Turkey

## Abstract

**Background:**

The accurate diagnosis of abdominal tuberculosis usually takes a long time and requires a high index of suspicion in clinic practice. Eighty-eight immune-competent patients with abdominal tuberculosis were grouped according to symptoms at presentation and followed prospectively in order to investigate the effect of symptomatic presentation on clinical diagnosis and prognosis.

**Methods:**

Based upon the clinical presentation, the patients were divided into groups such as non-specific abdominal pain & less prominent in bowel habit, ascites, alteration in bowel habit, acute abdomen and others. Demographic, clinical and laboratory features, coexistence of pulmonary tuberculosis, diagnostic procedures, definitive diagnostic tests, need for surgical therapy, and response to treatment were assessed in each group.

**Results:**

According to clinical presentation, five groups were constituted as non-specific abdominal pain (n = 24), ascites (n = 24), bowel habit alteration (n = 22), acute abdomen (n = 9) and others (n = 9). Patients presenting with acute abdomen had significantly higher white blood cell counts (p = 0.002) and abnormalities in abdominal plain radiographs (p = 0.014). Patients presenting with alteration in bowel habit were younger (p = 0.048). The frequency of colonoscopic abnormalities (7.5%), and need for therapeutic surgery (12.5%) were lower in patients with ascites, (p = 0.04) and (p = 0.001), respectively. There was no difference in gender, disease duration, diagnostic modalities, response to treatment, period to initial response, and mortality between groups (p > 0.05). Gastrointestinal tract alone was the most frequently involved part (38.5%), and this was associated with acid-fast bacteria in the sputum (p = 0.003).

**Conclusion:**

Gastrointestinal tract involvement is frequent in patients with active pulmonary tuberculosis. Although different clinical presentations of patients with abdominal tuberculosis determine diagnostic work up and need for therapeutic surgery, evidence based diagnosis and consequences of the disease does not change.

## Background

Tuberculosis (TB) is rarely seen in western countries, but it is not an uncommon disease in the developing world [1]. Its reappearance has increased in association with the acquired immunodeficiency syndrome (AIDS) and immigration to the United States [2-4]. TB in its various forms remains an important cause of morbidity and mortality in developing countries and in patients with AIDS [5].

Recently, along with the increased incidence of TB, extrapulmonary TB incidence has also increased [6,7]. The occurrence of abdominal TB is independent of pulmonary disease in most patients, with a reported incidence of coexisting disease varying from 5 to 36% [8]. In patients with abdominal TB, the highest incidence of disease was noted in the gastrointestinal tract and in the peritoneum, followed by the mesenteric lymph nodes. Within the gastrointestinal tract, the ileocecal area is the most common site of involvement [9].

In general, upper gastrointestinal (GI), hepatic and pancreatic involvements are rare in autopsy series and case reports [10-12].

Because of chronic and nonspecific clinical and radiological findings mimicking several diseases, such as Crohn's disease, carcinoma, sarcoma [13], amebiasis, yersinia infection, gastrointestinal histoplasmosis, and periappendiceal abscess [9], the diagnosis of the abdominal TB requires a high index of suspicion and, despite using newly developed diagnostic tools, it usually takes a long time to get accurate diagnosis in clinic practice. The patients' subjective complaints on admission should guide the diagnostic procedures [14].

In this report, the effect of different clinical presentations of abdominal TB on clinical course, diagnosis and outcome of the disease in 88 immune-competent patients who were grouped according to the gastrointestinal symptoms at admission and followed prospectively between 1995 and 2004.

## Methods

Patients, who were suspected to have abdominal tuberculosis through symptoms and / or operative findings, were prospectively evaluated. In addition to physical examination, basic laboratory studies, and anti-human immunodeficiency virus (HIV) serology, patients admitted with abdominal symptoms including pain, distension, nausea, vomiting, altered bowel habit, weight loss were evaluated for tuberculosis. Previous and family histories of TB were asked; chest X-ray, abdominal plain graph, tuberculin skin test and abdominal ultrasound (US) were routinely obtained during a nine-year period between 1995–2004. In case of suggestion of pulmonary TB in chest x-ray (infiltration or consolidation, nodularity, calcification, cavitary lesion, and fibrocalcific scar in lung parenchyma, pleural effusion, hilar or mediastinal lymphadenopathy), acid-fast bacilli (AFB) was searched in sputum and/or gastric juice. Sputum culture, and computed tomography (CT) of thorax were obtained. In case of negative results, bronchoscopy, and bronchoalveolar lavage plus biopsy were performed. If there was mediastinal lymphadenopathy (LAP) and the exact diagnosis could not be established by other means, mediastinoscopy with LAP biopsy was performed.

Based upon the clinical presentation, the patients were divided into five groups to guide diagnostic evaluations.

Group I: Patients with non-specific abdominal pain

Group II: Patients with ascites

Group III: Alteration in bowel habit (diarrhea, constipation or together)

Group IV: Acute Abdomen

Group V: Others (jaundice, dysphagia, fistula, fever of unknown origin, mass, weight loss)

Group I patients: abdominal CT, and according to the findings of CT, fine needle aspiration (FNA) biopsy or laparoscopy, upper and lower GI system endoscopy, and endoscopic biopsy for microbiologic and histopathologic diagnosis if a lesion seen.

Group II patients: ascitic cell count and type of cells, biochemistry analysis, smear for AFB, culture, IS*6110*-based in-house polymerase chain reaction (PCR) protocol for mycobacterium tuberculosis, and cytologic examination of ascitic fluid and abdominal CT.

Group III patients: colonoscopy and small bowel series, stool culture and AFB in stool if diarrhea present. Endoscopic biopsy for histopathologic examination and tissue culture and PCR for M. tuberculosis. Abdominal CT, and US. If ascites found, the exaimations in group II.

Group IV patients: histopathologic examination of surgical specimen if laparotomy performed. Abdominal CT, and colonoscopy if the patient is conservatively observed.

Group V patients: Upper endoscopy and thoraco-abdominal CT if dysphagia is present. Liver biopsy after excluding other causes if jaundice is present. Abdominal CT and if necessary MR and FNA in cases with abdominal mass were done. In patients with fistula only, AFB, culture and PCR of flux material; fistulography, CT, barium series or endoscopic examinations are performed to find out fistula tract.

Intraabdominal fluid (free or loculated; clear or complex with septae or debri), inter loop ascitis such as 'club sandwich' or 'sliced bread' signs which are due to localized fluid between radially oriented bowel loops, due to local exudation from the inflammed bowel, lymphadenopathy (discrete or conglomerated), bowel wall thickening and pseudo kidney sign were attributed to abdominal TB in abdominal US [15,16]. CT findings include adenopathy with low-density in centers, splenomegaly and hepatomegaly with nodules, ascites with complex features, bowel involvement, pleural effusion, intrasplenic, intrahepatic and intrapancreatic masses [8]. The colonoscopic features were defined as ulcers, nodules, deformed cecum and ileocecal valve, strictures, multiple fibrous bands and polypoid lesions [17].

Definitive diagnosis was based on one of the following methods with certain criteria: First of all, diagnostic priority for TB was given to show AFB microbiologically by culture or by both smear and PCR positivities, if that was not possible, the presence of caseating granulomatous lesions in the tissue was accepted as histopathological diagnosis of TB. If the diagnosis of TB was not obtained by using both of these methods, we achieved a clinical diagnosis of TB after a successful empiric therapeutic trial [9,18]. Empiric therapeutic trial was conducted for at least for 3 months with standard four drugs regiment (isonicotinic acid hydrazide, rifampin, pyrazinamide and ethambutol or streptomycin).

The following features were determined within each patient group: age, gender, disease duration, symptom frequency, TB presence in patient's history or environment, coexistence with pulmonary TB, tuberculin skin test status, laboratory findings, diseased segment within gastrointestinal segment, necessary diagnostic work-up, exact diagnosis methods, need for surgery except acute abdomen, response to therapy. The patients were followed up for adverse effects of medical treatment and surgical complications.

Numeric values were determined as percent or mean ± SD. Non-parametric Kruskal-Wallis test was used for quantitative results and chi-square test was used for qualitative results between groups. P value of less than 0.05 was considered to indicate the statistical significance.

## Results

After extensive clinical, endoscopic, radiologic, microbiologic and histopathologic investigation, we identified and followed 88 abdominal TB cases with gastrointestinal involvement. Age, gender, and symptoms on admission are summarized in Table 1; laboratory, chest X-ray, abdominal plain film and US findings are summarized in Table 2.

The most frequent symptom was abdominal pain (28.4%). TB was present in patient history and environment in 17 and 9.1 percent, respectively. Ascites was the most frequent physical finding (35.2%). Other physical examination findings were hepatomegaly (22.7%), splenomegaly (17%), abdominal mass (17%), fistula (12.5%), peripheral LAP (11.4%) and acute abdomen (10.2%). Most of the patients were significantly anemic. Erythrocyte sedimentation rate (ESR) and C-reactive protein (CRP) were mostly elevated in the study group. ESR was normal only in 4 cases (4.5%). Pulmonary active lesion or lesion consistent with prior TB were detected in 27.3 and 17 percent of the patients, respectively. There was significant association between AFB in sputum and GIS tract disease (p = 0.003). Ascites was detected in 40.9% of patients on US. On abdominal CT, besides ascites, gut wall thickness (frequently ileocecal region), LAP, abscess, and organomegaly etc. were detected in 81.8%.

According to clinical presentation, five groups were constituted as non-specific abdominal pain (n = 24), ascites (n = 24), alteration in bowel habit (n = 22), acute abdomen (n = 9), and others (n = 9). Age, gender, symptoms, need for surgery, diagnostic tests, how the diagnosis established (tissue and culture based against empiric), response to therapy according to the groups on admission are summarized in Table 3 and Table 4. With excluding grouping symptoms, mean white blood cell count (WBC) and lesion frequency in plain abdomen graphy were higher in patients admitting with acute abdomen (p = 0.002 and p = 0.014, respectively). Patients with alteration in bowel habit were statistically younger (p = 0.048). Abnormal finding frequency on abdominal CT was equal between groups (p = 0.196). CT guided FNA biopsy was preformed in 10 patients and its diagnostic yield was 70% in these patients. Colonoscopic lesion frequency was the lowest (7.5%) in colonoscopy-performed patients with ascites (p = 0.04). In our patients who had colonoscopic abnormalities, tissue culture and PCR positivity for TB was 11.1% and 19.2%, respectively. Although need for surgery rates was not different between patient groups, therapeutic surgery rate was significantly low (12.5%) in patients with ascites (p = 0.001). Gender, disease duration, associated symptoms, definitive diagnostic methods, response to therapy, time to response to therapy, and mortality were not different between groups (p > 0.05).

The localization and the type of disease were shown in Table 5. Gastrointestinal tract alone was the most frequent diseased part (38.5%) and iloececal region was the preferred area (53.8%) in the tract. Peritoneal and GI tract involvement proportions were approximately equal in patients admitted with abdominal pain. Isolated involvement of peritoneum was 71% in patients with ascites. The involvement rate of GI tract alone was 72.7% in patients with alteration in bowel habit. All patients with acute abdomen had GI tract involvement. Solitary mesenteric lymphadenitis (0.5%), liver (0.5%), pancreas (0.5%) and esophagus (0.5%) involvements were established.

In the whole group, tissue and culture based and empiric therapeutic trial diagnosis rates were 75.4 and 14.6 percent, respectively.

Peroperative mortality in 1 case, fistula in 3 cases, short bowel in 1 case, and chronic pancreatic insufficiency in 1 patient developed as complications.

## Discussion

Patients with abdominal TB may have many symptoms and mimic many diseases, therefore if it is not clinically suspected, it may result in important morbidity and mortality. In abdominal TB series GI tract and peritoneum are reported as the most frequent sites of involvement [9]. Four major pathophysiologic mechanisms are proposed for abdominal TB: hematogenous spread, swallowing of infected sputum, ingestion of contaminated milk or food, and contiguous spread from adjacent organs [19].

In our patients, GI tract alone, peritoneum alone, and GI tract with peritoneum involvement (multi-site) were most frequent and detected in 38.5, 28, and 20.4 percent, respectively. Mesenteric lymph node involvement alone was rare, liver involvement was uncommon, but even esophageal and pancreatic involvement mimicking tumor was present.

The ileocecal area is the most frequent site of involvement in the GI tract [9,20]. Physiologic stasis, absorption of digested materials, and augmented lymphoid tissue at ileocecal area may be the causes of this property [21,22]. In our patients, GI tract and ileocecal region in particular, was also most frequently diseased part.

Gastroenterological symptoms of abdominal TB depend on the organ or tissue involved. Abdominal pain, tenseness, and diarrhea, as our patients demonstrated, are frequent symptoms. Also, as reported in the literature, active TB lesion or lesion consistent with prior TB in the lungs are detected in 27.3 and 17 percent of the whole group of patients, respectively [9,18]. Diagnosis is difficult in the absence of pulmonary involvement. As a result, clinical and radiologic signs of pulmonary TB must be searched for in every patient even in the absence of pulmonary symptoms. Meantime, absence of pulmonary findings does not rule out abdominal TB.

As reported previously [14], subjective complaints of the patients could guide the diagnostic procedures. For this reason, to group the patients according to their symptoms on admission may cause the diagnostic work up to be more cost effective. When our patients are grouped according to symptoms, disease duration, gender, weight loss, fever, and previous or family history of TB were not different between groups. There was no significant difference in frequency of radiologic pulmonary lesions, tuberculin skin test status, and ESR or CRP elevation between groups. This observation has not been reported before.

Although WBC, and abdominal plain film abnormalities were higher in patients with acute abdomens, these findings were nonspecific for TB diagnosis. On the other hand, macroscopic findings and histopathologic and microbiologic investigations of the tissues obtained during therapeutic surgery made abdominal TB diagnosis easier and faster in this specific patient group. Abdominal CT abnormalities regarding TB were observed in all patients with acute abdominal findings. This feature has given an opportunity to follow these patients, excluding those with perforation, with conservative medical treatment [8,23]. In this subgroup of patients colonoscopy during follow-up revealed abnormality in 75%, and provided histopathologic and microbiologic TB diagnosis in 50%.

Although both abdominal US and CT are equally sensitive in detecting ascites, CT is superior to US in detecting LAP, gut wall thickness, omental cake, and other abnormalities [24,25]. Abnormal abdominal CT findings in our series were higher than US findings and were equal in each group. CT shows localization and type of the lesion, but it is not specific for TB diagnosis [26,27]. CT guides the following diagnostic methods such as FNA, endoscopy, laparoscopy or laparotomy [16,27].

Therapeutic and diagnostic surgery rates were similar between our patient groups. This may be due to two reasons. Firstly, ascites examination, FNA biopsy, and endoscopic methods may be inadequate for definitive diagnosis. Secondly, patients with abdominal pain or altered bowel habit groups may develop complications requiring surgery. Diagnostic laparotomy was needed most often in patients with ascites. Infrequent detection of AFB in ascitic fluid, late results of ascites cultures, and uncommon culture yield of M. tuberculosis are reasons for increased laparoscopy/laparotomy need [28,29]. Diagnostic operation was lower in patients with alteration in bowel habit and the group in which GI tract was not directly involved. Endoscopic examinations increased diagnostic efficiency and decreased diagnostic surgical interventions in patients with alteration in bowel habit. Patients with acute abdominal symptoms frequently have GI tract and particularly ileocolonic involvement. Colonoscopy is effective to establish TB diagnosis in patients that could be managed medically in this group. Colonoscopy provides tissue for culture, PCR and histopathologic examination [30]. In our patients who had colonoscopic abnormalities, tissue culture and PCR positivity for TB rate was lower than or at most equal to previously reported series [17,31,32]. Low colonoscopic biopsy count and sample size may affect this result. Stool examination for AFB and culture for TB are proposed in the literature, but the association of GI TB with AFB in the sputum may cause misinterpretation [19].

AFB is searched for in the sputum and when necessary in gastric juice in our patients with pulmonary involvement. There was a positive association between AFB positivity in sputum and GI tract involvement. This association can be explained with swallowing infected sputum [19]. AFB in sputum rate was not different between groups; this can be explained by existence of GI tract involved patients in groups formed with patient symptomatology. Patients with alteration in bowel habit were younger as reported in the literature. Wig K and et al. reported that the maximum age incidence of intestinal tuberculosis was also 15 to 24 years [33]. The reason of the high incidence rate of intestinal tuberculosis in young population could be related to Peyer's patches which play a major role in intestinal immunity and are portals of entry for significant pathogens. Peyer's patches in the small intestine peaks at ages 15–25 and then declines with age [34].

There was no significant difference between evidence based diagnosis and response to therapy diagnosis in all groups.

Our diagnosis rate based on response to therapy was similar to the rates reported before [18]. There was no difference in evidence based and response to treatment diagnosis between our groups. There was also no difference in first response time to therapy between two diagnostic methods. Empiric therapeutic trial appears to be a useful method for rapid presumptive diagnosis and treatment of tuberculosis [35].

It is reported that there is no difference in response or relapse rates between 9 month and 18 month treatment times [36]. All our patients received therapy for 9 months and had a high cure rate. High cure and low relapse rates may be caused by immune competence of our patients and infrequency of primary drug resistance in this group. High adherence to therapy rate and low adverse effects of drugs to lower dose rate may influence secondary drug resistance.

Fistula, short bowel syndrome, and anastomosis leak are frequent complications of surgery performed in abdominal TB [37]. Our patients had similar complications.

## Conclusion

Gastrointestinal tract involvement is frequent in patients with active pulmonary tuberculosis. Different clinical presentation of patients with abdominal TB involving GI system may determine the necessity of diagnostic or therapeutic surgical interventions, but evidence based diagnosis and prognosis are not influenced.

## Abbreviations

TB, tuberculosis; AIDS, acquired immunodeficiency syndrome; GI, gastrointestinal; HIV, human immunodeficiency virus; US, ultrasound; AFB, acid-fast bacilli; CT, computed tomography; LAP, lymphadenopathy; PCR, polymerase chain reaction; FNA, fine needle aspiration; ESR, erythrocyte sedimentation rate; CRP, C-reactive protein; WBC, white blood cell count;

## Competing interests

The author(s) declare that they have no competing interests.

## Authors' contributions

CB conceived of the study, and participated in its design and coordination. FFB, TK, NA, HMS, ATI, MG, EC and GK collected the samples and clinical data, and carried out the laboratory analysis. ARD and FFB conceived of the study and participated in the sequence alignment and drafted the manuscript. OO participated in study design and coordination and revised the manuscript critically for important intellectual content. All authors read and approved the final manuscript.

## Pre-publication history

The pre-publication history for this paper can be accessed here:



## Figures and Tables

**Table 1:** Demographic and clinic features in whole group.

Patients, (n)	88
Age (year)	31.4 ± 15

Gender (F/M)	48/40

Disease duration (month)	10.4 ± 19

Main symptom (n/%)	
Abdominal pain	25 (28.4%)
Abdominal distension	23 (26.1%)
Diarrhea	15 (17%)
Fever	4 (4.5%)
Weight loss	4 (4.5%)
Ileus	4 (4.5%)
Perforation	4 (4.5%)
Others	4 (4.5%)
Dysphagia	2 (2.3%)
Icter	2 (2.3%)
Dyspepsia	1 (1.1%)

Previous history for TB	15 (17%)

Family history for TB	8 (9.1%)

F, Female; M, Male; TB, Tuberculosis.

**Table 2:** The results of the diagnostic methods in whole group.

ESR (mm/h)	68 ± 24
CRP (mg/L)	53 ± 39

WBC (/mm^3 ^× 1000)	9.2 ± 3.6

Hct %	32.3 ± 5.2

Hb (g/dL)	10.6 ± 1.9

Chest X-ray %	
Active lesion	24 (27.3%)
Lesion consistent with prior TB	15 (17%)
Normal	49 (55.7%)

AFB positivity in sputum %	11 (12.6%)

Lesion frequency in abdominal plain graphs %	21 (23.9%)

Abdominal US abnormality*	78 (88.6%)

*Including hepatomegaly-splenomegaly

ESR, erythrocyte sedimentation rate; CRP, C-reactive protein; WBC, white blood cell count;

Hct, haemotocrit; Hb, haemoglobin; AFB, acid-fast bacteria; US, ultrasonography.

**Table 3:** Demographic and clinical features in divided groups based upon the clinical presentation.

Groups*	I (n = 24)	II (n = 24)	III (n = 22)	IV (n = 9)	V (n = 9)	P
Age (yr)	36.5 ± 20	30.4 ± 13	23.5 ± 8	32.7 ± 12	38 ± 16	0.048
Gender (F/M)	12/12	13/11	14/8	2/7	5/4	0.34
Disease Duration (mo)	11.3 ± 23	6 ± 8	9.8 ± 14	25 ± 41	6.6 ± 5	0.869
Previous history of TB %	16.7	8.3	18.2	22.2	33.3	0.530
Family history for TB %	8.3	4.2	18.2	11.1	0	0.425
Weight loss (kg)	8.2 ± 7	6 ± 4.5	8.5 ± 6	8.7 ± 5	8.1 ± 7.2	0.496
Temperature (°C)	37.5 ± 1	37.4 ± 0.8	37.5 ± 1	37.9 ± 0.9	37.2 ± 1	0.615
Fistula (%)*	8.3	0	31.8	0	22.2	0.07
Therapeutic Operation %	33.3	12.5	27.3	66.6	22.2	0.001
Response to therapy %	91.7	95.8	90.9	77.7	100	0.354
First response to therapy (mo)	2.5 ± 1.5	2.1 ± 1.6	1.9 ± 1.3	2.6 ± 1.5	2.8 ± 2	0.298
Death %	8.3	4.2	4.5	11.1	0	0.825

*Group I, non-specific abdominal pain; Group II, ascites; Group III, alteration in bowel habit (diarrhea, constipation or together); Group IV, acute abdomen; Group V, others (jaundice, dysphagia, fistula, fever of unknown origin, mass, weight loss);

Yr, year; F, female; M, male; Mo, month; TB, tuberculosis.

P value of less than 0.05 was considered to indicate the statistical significance in between groups.

**Table 4:** The results of the diagnostic laboratory methods, the rate of abnormality in imaging and invasive methods, and definitive diagnosis in divided groups based upon the clinical presentation.

Groups*	I (n = 24)	II (n = 24)	III (n = 22)	IV (n = 9)	V (n = 9)	P
WBC (/mm3 × 1000)	10 ± 4	8.7 ± 2.6	7.7 ± 1.8	14 ± 3.7	7.3 ± 2.8	0.002

ESR(mm/h)	67 ± 21	65 ± 31	71 ± 20	69 ± 21	72 ± 26	0.842

Tuberculin skin test (mm)	17 ± 7	18 ± 6.5	13 ± 7	18 ± 11	16 ± 9	0.119

AFB in sputum %	12.5	4.5	18.2	25	11	0.602

Chest X-ray %	54.2	27.5	40.9	66.6	22.2	0.448

APG %	20.8	12.5	31.8	66.7	0	0.014

Abdominal CT %	91.7	100	90.9	100	77.8	0.196

** Colonoscopy %	62.5 (5/8)	7.5 (2/15)	90.9 (20/22)	75 (3/4)	33.3 (1/3)	0.04

Operation %	54.2	54.2	33.3	75	22.2	0.156

Diagnosis %						
Evidence based	79.2	83.3	86.4	88.9	88.9	
Histopathologic	62.5	50	45.5	44.4	55.6	
Microbiologic	4.2	12.5	9.1	33.3	11.1	0.933
H+M	12.5	20.8	31.8	11.1	22.2	
ETT%	20.8	16.7	13.6	11.1	11.1	

*Group I, non-specific abdominal pain; Group II, ascites; Group III, alteration in bowel habit (diarrhea or constipation or together); Group IV, acute abdomen; Group V, others (jaundice, dysphagia, fistula, fever of unknown origin, mass, weight loss);

** The percent in patients with colonoscopy performed WBC, white blood cell count; ESR, erythrocyte sedimentation rate; AFB, acid-fast bacteria; APG, abdominal plain graphy; CT, computed tomography; H, histopathologic; M, microbiologic; ETT, empiric therapeutic trial. P value of less than 0.05 was considered to indicate the statistical significance in between groups.

**Table 5:** The distribution of involvement area in the groups*.

Localization	Group I (n = 24)	Group II (n = 24)	Group III (n = 22)	Group IV (n = 9)	Group V (n = 9)	Total (n)
Multiple (n) (Peritoneum, SB, colon, pericardium)	2	7	6	1	2	18

GI Tract (n)						34
SB			3	2	1	
Ileocecal	6		7	5		
Colon			6	1		
Stomach	1					
Esophagus					2	

Liver	3				1	4

Peritoneum	8	17				25

Mesenteric LAP	3				2	5

Pancreas and biliary system	1				1	2

Total (n)	24	24	22	9	9	88

*Group I, non-specific abdominal pain; Group II, ascites; Group III, alteration in bowel habit (diarrhea or constipation or together); Group IV, acute abdomen; Group V, others (jaundice, dysphagia, fistula, fever of unknown origin, mass, weight loss);

SB, small bowel; GI, gastrointestinal; LAP, lymphadenopathy
